# Three-dimensional Bragg coherent diffraction imaging of an extended ZnO crystal^1^

**DOI:** 10.1107/S0021889812018900

**Published:** 2012-06-20

**Authors:** Xiaojing Huang, Ross Harder, Steven Leake, Jesse Clark, Ian Robinson

**Affiliations:** a London Centre for Nanotechnology, University College London, London WC1H 0AH, UK; b Advanced Photon Source, Argonne National Laboratory, Argonne, IL 60439, USA; c Research Complex at Harwell, Didcot, Oxfordshire OX11 0DE, UK

**Keywords:** three-dimensional quantitative imaging, coherent diffraction imaging, ptychography, zinc oxide

## Abstract

A complex three-dimensional quantitative image of an extended zinc oxide (ZnO) crystal has been obtained using Bragg coherent diffraction imaging integrated with ptychography. By scanning a 2.5 µm-long arm of a ZnO tetrapod across a 1.3 µm X-ray beam with fine step sizes while measuring a three-dimensional diffraction pattern at each scan spot, the three-dimensional electron density and projected displacement field of the entire crystal were recovered. The simultaneously reconstructed complex wavefront of the illumination combined with its coherence properties determined by a partial coherence analysis implemented in the reconstruction process provide a comprehensive characterization of the incident X-ray beam.

## Abstract 2

A complex three-dimensional quantitative image of an extended zinc oxide crystal has been obtained using Bragg coherent diffraction imaging integrated with ptychography.

## 1. Introduction

Coherent diffraction imaging (CDI) is a rapidly developing microscopy technique which can provide nondestructive two- and three-dimensional images of materials (Miao *et al.*, 1999 ▶; Chapman *et al.*, 2006 ▶) and biological samples (Shapiro *et al.*, 2005 ▶; Jiang *et al.*, 2010 ▶) with the potential of reaching diffraction-limited resolution. This method replaces the optical lens of a traditional microscope with iterative reconstruction algorithms, which recover unique real-space images (Bates, 1982 ▶) through phase retrieval using information encoded in oversampled far-field diffraction intensities. In order to meet the stringent sampling requirement, the application of CDI was limited to either finite samples isolated inside the illumination or a finite sample area illuminated by a collimated beam (Abbey *et al.*, 2008 ▶). To remove this size constraint, speed up algorithm convergence and provide artefact-free images, phase-diverse methods were developed to utilize multiple measurements on a common sample section with a set of diverse illuminations (Putkunz *et al.*, 2011 ▶). Ptychography (Rodenburg *et al.*, 2007 ▶) provides one of those phase diversities by scanning an extended sample through a confined illumination. The redundant information in coherent diffraction data from overlapped scanning positions releases the oversampling criterion. This redundancy also makes it possible to recover the complex wavefront of the illumination beam simultaneously (Thibault *et al.*, 2008 ▶).

Combining ptychography with Bragg CDI (Pfeifer *et al.*, 2006 ▶) allows investigation of strain fields inside crystalline samples with arbitrary sizes (Godard *et al.*, 2011 ▶). The use of diffraction data surrounding a crystal Bragg peak instead of just the forward scattering renders the CDI method sensitive to strain (Robinson & Harder, 2009 ▶). We used this combined approach by translating an extended crystalline sample across a focused X-ray beam. By inverting a series of three-dimensional diffraction patterns measured at scanning positions, we obtained the three-dimensional electron density and dis­place­ment field distribution of the extended crystalline sample. Considering that X-ray beams from a third-generation synchrotron are partially coherent while CDI assumes that the sample is coherently illuminated, we adapted recently developed algorithms to accommodate partial coherence in the reconstruction process (Clark *et al.*, 2011 ▶). The obtained coherence properties and reconstructed beam wavefront provide a comprehensive characterization of the incident X-ray beam.

We have imaged sections of ZnO wires with a confined X-ray beam in our previous study with the traditional single-step Bragg CDI method (Xiong *et al.*, 2011 ▶). In that work, the notable result was the observation of rough ends on the wire sections. Because the image boundary was defined by the size of the beam, and the beam intensity can drop to a low level at its edges, the image boundary may not be well determined. As a result, this weak illumination at the edges causes roughness. The incident beam profile cannot be factorized without the provision of extra information through diversity. This problem can also be solved by the newly conducted ptychographical measurement, which provides such additional information and allows the separation of the sample structure from that of the beam illumination.

## 2. Experiment

The sample we studied was a ZnO tetrapod. ZnO has been widely used as an industrial material because of its tunable conductivity through doping (Davies *et al.*, 2009 ▶), as well as its piezoelectric (Song *et al.*, 2006 ▶) and pyroelectric nature (Hsiao *et al.*, 2008 ▶). The ZnO structure was synthesized (Leake, 2010 ▶) by the chemical vapor transport deposition method (Newton *et al.*, 2006 ▶). A mixture of zinc carbonate and graphite powder was heated to 1173 K with argon carrier gas flowing through the furnace. The ZnO crystals formed in the vapor phase, then landed on a silicon substrate placed downstream of the argon gas flow. The sizes and shapes of the obtained crystals could be controlled by the ratio of zinc carbonate and graphite powder in the mixture, the heating temperature in the furnace, the carrier gas flow rate, and the surface temperature of the silicon substrate. The synthesized ZnO crystals can form a wide range of morphologies such as hexagonal plates, nanowires and tetrapods (Wang, 2004*a* ▶).

**Figure 1:**
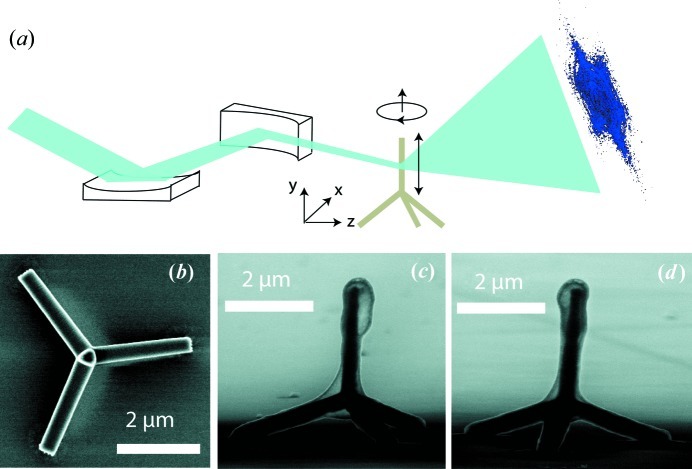
(*a*) Schematic illustration of the experimental setup. (*b*) Top-view SEM image of the ZnO tetrapod taken one day before the X-ray experiment. (*c*), (*d*) Side-view SEM images of the same ZnO tetrapod taken two years after the X-ray measurement with 30 keV electrons, where a condensed carbon layer was found on the sample surface. The width and length of the vertical leg are about 0.37 and 2.5 µm, respectively.

The ZnO tetrapod has four wurtzite ZnO arms joined at a central zinc blende core (Newton & Warburton, 2007 ▶). The wurtzite arms tend to maximize {211} or {011} facets, believed to lower their surface energy (Wang, 2004*b* ▶). The sample we measured in this experiment is a vertically standing arm of a ZnO tetrapod with the other three arms bonded to a silicon substrate. The arms of this ZnO tetrapod have {011} side facets that encircle a triangular cross section as shown in Fig. 1 ▶(*b*), which was taken a day before the X-ray experiment. Side-view scanning electron microscopy (SEM) images (Figs. 1 ▶ *c* and 1 ▶ *d*) were taken from the same tetrapod two years after the X-ray measurement. We found that the sample was covered by a layer of relatively light elements, which is semi-transparent with a 30 keV electron beam. An energy dispersive spectroscopy measurement showed that the layer is mainly carbon. This side-view image also shows that the width and length of the vertical arm are 0.37 and 2.5 µm, respectively.

The ptychographical Bragg CDI experiment was performed at Beamline 34-ID-C of the Advanced Photon Source at Argonne National Laboratory. The ZnO tetrapod was mounted in the rotation center of the diffractometer with the vertical arm pointing straight up. A CCD detector with 22.5 µm pixel size was located 1.5 m away and oriented at the 101 Bragg peak. The three-dimensional diffraction pattern was collected by rotating the tetrapod from −0.25 to +0.25° along the vertical direction with 0.01° angular step size. At each rotation angle, 50 accumulated measurements were collected with 0.1 s exposure time. Coherent 11.5 keV X-rays from an undulator were focused by Kirkpatrick–Baez (KB) mirrors to about 1.5 × 1.5 µm. This focus size was estimated by scanning a tungsten wire cross through the beam, which acted as a knife edge. The derivative of the obtained transmission signal was then fitted with a Gaussian function giving the beam size. This measurement convolves the beam size with the shape of the wire, so that the actual focus size is slightly overestimated.

The vertical beam source size 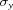 of the beamline is determined by the electron beam size in the undulator, which is typically 40 µm (Robinson *et al.*, 2003 ▶). The distance from the undulator to the illumination-defining aperture (roller blade slits) is about 54 m. The vertical coherence length, defined as the half-width at half-maximum (HWHM) of the mutual coherence function, can be estimated as (Nugent, 2010 ▶)
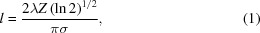
where 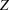 is the distance from the source. With 11.5 keV X-rays, 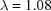
 Å, the vertical coherence length at the roller blade slits is about 72 µm. The typical roller blade setting is 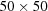
 µm, so that the aperture is coherently illuminated in the vertical direction. This full vertical coherence was maintained by KB mirrors to the focus spot. The horizontal beam source 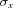 is set to 150 µm by a source-defining slit, which is placed about 27 m before the roller blades. Using equation (1), we can estimate that the horizontal coherence length at the roller blade is about 10 µm, which is one-fifth of the roller blade slit’s horizontal opening. This ratio is conserved in the 1.5 µm focus (Vartanyants & Singer, 2010 ▶), which gives 300 nm horizontal coherence at the focus. The longitudinal coherence length of the same beamline was previously measured to be 660 nm (Leake *et al.*, 2009 ▶). Compared with the dimension of the ZnO tetrapod arm, we found that the horizontal coherence length is slightly smaller than the sample width. Since the coherence lengths of the illumination were of similar size to the object, the assumption that the illumination is fully coherent is not strictly correct, and we implemented an algorithm (Clark *et al.*, 2011 ▶) that handles partial coherence in the data reconstruction process.

The tetrapod was translated through the beam along the vertical direction in six steps with step size less than 0.5 µm. At each scan position, three-dimensional diffraction data were collected with a ±0.25° rocking rotation and 0.01° step size, as shown in Fig. 1 ▶(*a*). The cropped and binned effective array size for each scan is 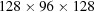
 pixels. This arrangement gives a real-space voxel size of 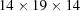
 nm.

The ZnO arm extends only in the vertical direction, while its sizes in the other two directions are smaller than the incident X-ray beam. The rotation axis for rocking-curve measurement is along the vertical direction as well, so that this setup ensures that the X-ray beam illuminates the same section of the ZnO arm during three-dimensional data acquisition. Since the ptychographical scan is only performed vertically, it is expected that the reconstructed beam wavefront will contain complete information only along its vertical direction, and partial information in the horizontal direction within the width of the sample.

## 3. Results

### 3.1. Individual reconstruction

**Figure 2:**
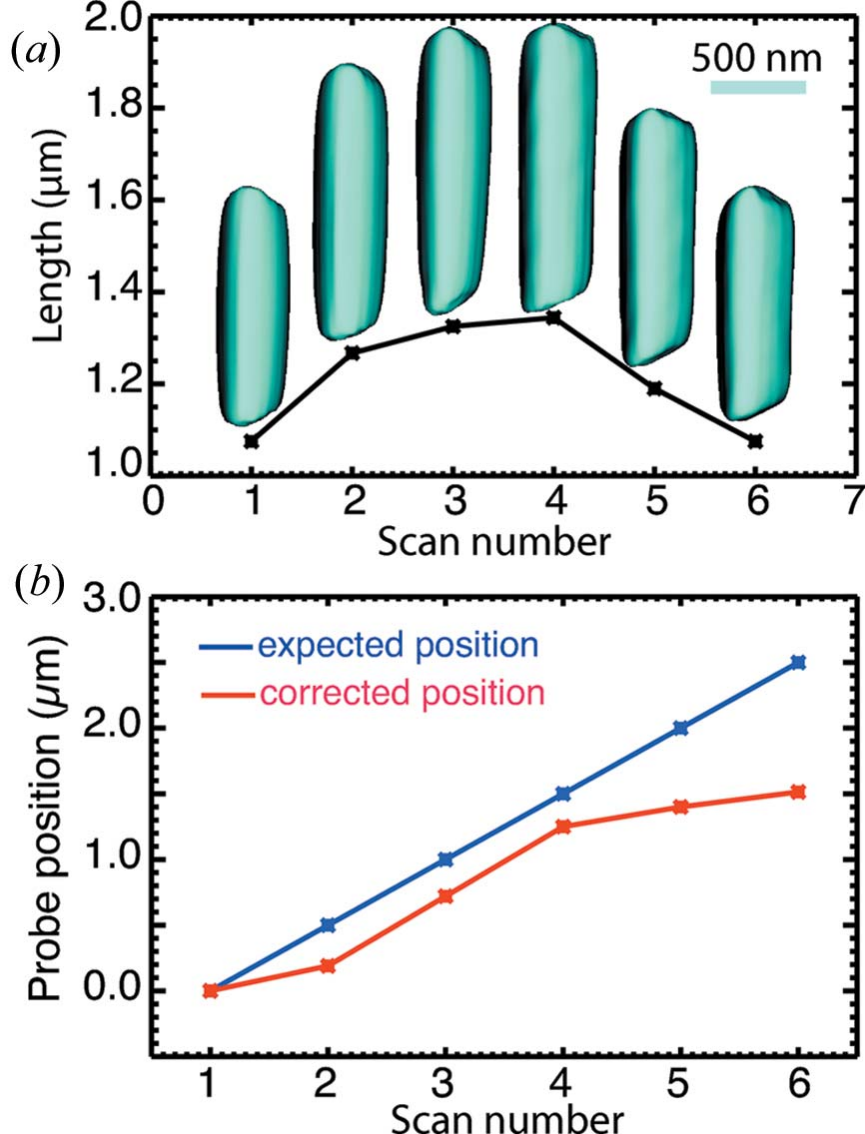
(*a*) Plot of the observed lengths of 20% isosurfaces of the reconstructed magnitude from individual measurement at each scan position. (*b*) Plots of expected probe positions and corrected probe positions using reconstructed images.

Because a full three-dimensional diffraction pattern was measured at each ptychographical scan position, each data set can be reconstructed individually using regular CDI algorithms to give a three-dimensional image of the illuminated sample section. Although these individually reconstructed images (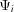) are products of the object section (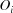) and the complex incident beam or probe (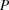), they can give a good estimation of the length for the illuminated section at each scan position. The magnitude isosurfaces and the lengths of the reconstructions of six data sets are plotted in Fig. 2 ▶(*a*). The length plot peaks in the center, which is consistent with the experimental sequence of scanning the ZnO arm across the X-ray beam from top to bottom, since the X-ray beam intersects the most at the center and moves off the sample at both ends. The section length of each individual reconstruction also provides information on how much the vertical motor moved at each step, which was used to compensate for the positioning accuracy of the vertical step motor used in this experiment (shown in Fig. 2 ▶ *b*).

The obtained images from single data set reconstructions show triangular cross sections with a width of about 370 nm, which agree with SEM measurements of the same tetrapod. The maximum section length is 1.3 µm, which implies that the focused beam size is about 1.3 µm in the vertical direction.

### 3.2. Modified ptychographical algorithm

Since the reconstruction result from each individual data set is the product of object and probe (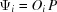), it is equivalent to and can replace the product-updating process in the ptychography algorithm (Thibault *et al.*, 2008 ▶). The reconstruction sequence for each single data set comprised ten error reduction (ER) cycles and 150 hybrid input–output cycles, followed by another 40 ER algorithm cycles. The shrink-wrap algorithm (Marchesini *et al.*, 2003 ▶) was applied to refine the support for each section. The first round of individual reconstructions assumes a uniform plane wave illumination and a random starting guess of the object. With the *n*th-round reconstruction results, 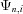, the probe is updated as
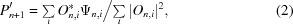
where 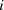 denotes the *i*th scan spot and 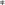 denotes the complex conjugate. The obtained probe 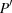 is a three-dimensional complex array. In this experiment, the sample thickness is much smaller than the depth of focus of the KB mirrors, so we can approximate the probe as a two-dimensional wavefront whose variation in the 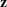 direction is negligible when propagating through the sample. 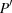 is converted to a two-dimensional probe by
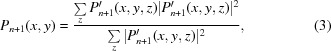
where 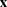 and 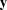 are directions transverse to the propagation direction 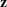. A new three-dimensional probe 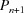 is generated by setting each frame in the 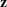 direction to 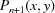.

Then, the object can be updated as
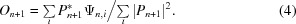
Finally, the product 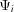 is updated and used as the starting image for the next round of individual data set reconstructions:
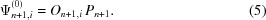
This reconstruction process differs from the original proposed ptychographical iterative engine (Rodenburg *et al.*, 2007 ▶), which does not update the probe, and also differs from the extended algorithm (Thibault *et al.*, 2009 ▶; Maiden & Rodenburg, 2009 ▶), which updates the image view in the same iterative cycle and simultaneously recovers the probe function. Here, the product of object and probe is updated from independent reconstructions of individual data sets.

### 3.3. Sample reconstruction

**Figure 3:**
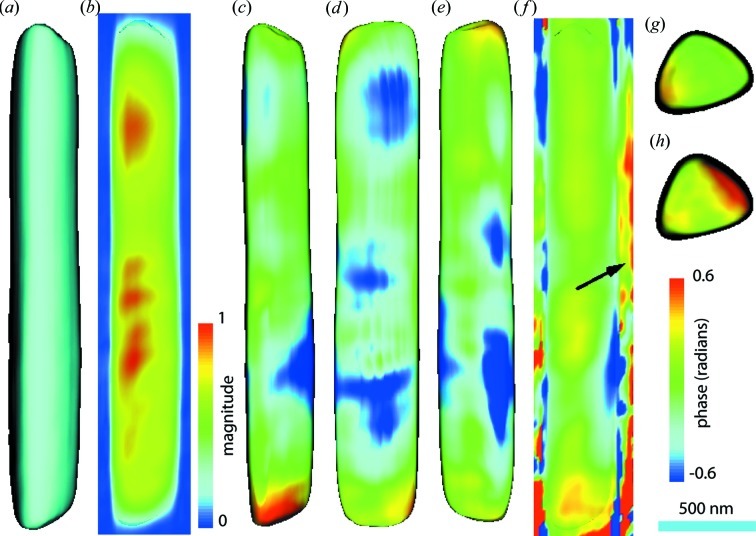
20% isosurface (*a*) and central cut plane (*b*) of the ptychographical reconstructed magnitude. (*c*), (*d*), (*e*) Phase distribution on three side facets of the 20% magnitude isosurface. (*f*) Central cut plane of the phase image, showing the 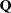 vector. (*g*), (*h*) Phases on top and bottom surfaces.

We ran ten rounds of single data set reconstructions and updating probe/object/product cycles. The obtained object and probe were stabilized after five rounds. The image of the entire ZnO vertical arm is shown in Fig. 3 ▶.

Fig. 3 ▶(*a*) shows that the length of the obtained ZnO arm is 2.57 µm, and its width is about 0.37 µm, both of which are consistent with expectations from SEM images. The slightly narrower top-half section as seen in Fig. 1 ▶(*d*) is also represented well in the reconstructed image. The central cut plane of the obtained magnitude as shown in Fig. 3 ▶(*b*) is smoother than what we observed before with regular CDI (Xiong *et al.*, 2011 ▶); however, unphysical density modulations still persist, artefacts that have been previously referred to as ‘hot spots’ (Vartanyants & Robinson, 2001 ▶; Leake *et al.*, 2009 ▶). This effect may be caused by the incomplete recovery of the probe in the horizontal direction.

Figs. 3 ▶(*c*)–3 ▶(*e*) display the phase distributions on three side facets. The phase varies in a small range from −0.6 to +0.6 radians, which indicates that this ZnO arm is only slightly strained. We found the strongest phases located near the bottom of the arm, as shown in Figs. 3 ▶(*c*) and 3 ▶(*h*). Considering that the wurtzite arm’s (001) bottom plane connects the zinc blende core’s (111) top plane (Manna *et al.*, 2003 ▶) directly at the bottom of the arm, the lattice mismatching might raise local strain near the interface and relax towards the arm. The central cut plane of phases (Fig. 3 ▶ *f*) also shows moderate phase distribution inside the crystal body and large phases near the bottom.

The top and bottom surfaces of the ptychographically reconstructed image (Figs. 3 ▶ *g* and 3 ▶ *h*) are much smoother than what we obtained previously with regular CDI (Xiong *et al.*, 2011 ▶). Factorizing the beam profile from the object structure removes the artefacts introduced by the beam and improves the magnitude fluctuations at the sample edges. Implementing partial coherence analysis of the beam also removes roughness in the obtained images, which can be seen in reconstructions with single data sets (Fig. 2 ▶).

### 3.4. Probe reconstruction

**Figure 4:**
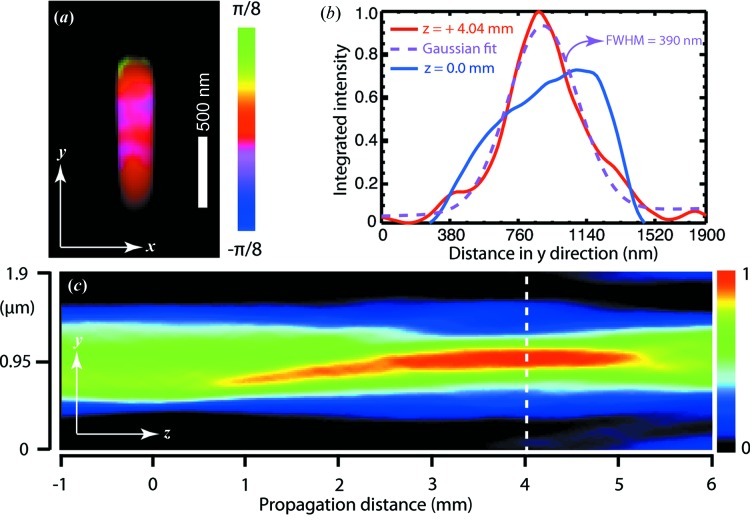
(*a*) Reconstructed complex wavefront of the probe in the sample plane, with brightness and hue illustrating magnitude and phases, respectively. (*b*) Line plot of beam intensity integrated along the **x** direction at 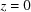
 mm and 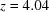
 mm. (*c*) Beam intensity integrated along the **x** direction of the propagated probe. The vertical focus is located 4.04 mm downstream of the sample plane.

The reconstructed complex wavefront of the X-ray beam (the probe) is shown in Fig. 4 ▶(*a*), where the brightness shows the magnitude and the color map shows the phase of the probe. The vertical length of the probe is about 1.3 µm as expected. There is not much phase curvature in the probe at the sample plane. The phase distribution inside the probe spans only a small range between 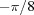 and 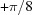. A phase variation around zero can be barely seen in the probe, while a noticeable strong phase stripe can be recognized at its top.

Since the ptychographical reconstruction gives the complex wavefront of the X-ray beam at the sample plane, we can propagate this wavefront to any plane along its propagation direction (Nugent, 2010 ▶). Since a one-dimensional ptychographical measurement was performed in this experiment, there is no redundant overlapping information in the horizontal direction. As a result, we only recovered the part of the probe that illuminated the ZnO sample in the horizontal direction; thus the propagated image might not be meaningful in the horizontal direction. The wavefront in the vertical direction was fully reconstructed. Fig. 4 ▶(*c*) shows the integrated intensity along the 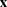 direction at different propagation distances. We found that the vertical focal plane was located about 4 mm downstream of the sample plane. The focal plane is taken to be the place at which the propagated wavefront reaches the highest intensity and the narrowest width (as shown in Fig. 4 ▶ *b*). A Gaussian fit gives a full width at half-maximum of 390 nm at the vertical focal plane. This 4 mm defocus distance is extremely difficult to detect with conventional focus-checking methods, such as scanning a tungsten wire. Using the ptychographical reconstruction methods, we observed a slight phase curvature of the recovered probe in the sample plane, which provides evidence of misfocusing and can be confirmed by wavefront propagation.

### 3.5. Coherence recovery

**Figure 5:**
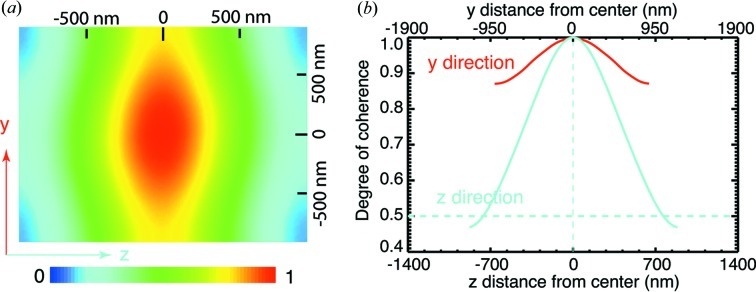
(*a*) The central 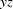 frame of the recovered coherence function. (*b*) Plots of the central lines in the 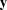 and 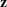 directions of the coherence function. The coherence in the 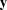 direction is good, above 0.8. The half-width of the 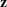-direction line plot at 0.5 gives an HWHM coherence length of 720 nm.

Another important X-ray beam property for CDI experiments is the coherence. X-ray beams from third-generation synchrotron undulators are partially coherent (Paterson *et al.*, 2001 ▶). As a result, the measured far-field diffraction intensity is a convolution of the diffraction pattern of the sample and the Fourier transform of the mutual coherence function (Nugent, 1991 ▶), which degrades fringe or speckle visibility. Because the CDI phase retrieval algorithms assume a fully coherent illumination, the partial coherence nature of the incident X-ray beam causes artefacts in reconstructed images (Vartanyants & Robinson, 2003 ▶). Newly developed algorithms adapt to spatial (Whitehead *et al.*, 2009 ▶; Clark & Peele, 2011 ▶) and temporal (Chen *et al.*, 2009 ▶; Abbey *et al.*, 2011 ▶) partial coherence, and mitigate artefacts in the obtained images. We utilized the method proposed by Clark & Peele (2011 ▶) and extended it into three-dimensional cases, so that the three-dimensional coherence function of the incident beam can be recovered simultaneously by implementing partial coherence analysis in the reconstruction process (Clark *et al.*, 2011 ▶). We integrated the same algorithm into the reconstructions with single data sets. As shown in Fig. 2 ▶, the reconstructed images with single data sets are improved compared with our previous study. The recovered coherence function in the central *yz* plane is shown in Fig. 5 ▶(*a*). The central line plot of the vertical coherence function is above 0.8, which implies the coherence property is very good in the measurement range according to the Rayleigh criterion (Born & Wolf, 1999 ▶), and this agrees with the coherence length estimation. When the central line plot in the 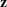 direction drops to 0.5, it gives an HWHM coherence length of 720 nm, which is consistent with previous measurements of 660 nm (Leake *et al.*, 2009 ▶).

## 4. Discussion

For an object with extended dimensions in two directions, a two-dimensional ptychographical scan is needed to provide sufficient field of view. The two-dimensional scan pattern also gives sufficient overlapping information to recover the full two-dimensional complex wavefront of the probe. It has been considered that the periodicity in the raster scan steps can introduce artefacts in the obtained image (Thibault *et al.*, 2009 ▶), and irregular scan patterns were suggested for ptychographical measurements. Here, we performed a one-dimensional ptychographical scan with scan steps varying around 0.5 µm, which breaks the periodicity of the scan pattern.

A density modulation was observed in the reconstructed magnitude, which was also observed in related work (Godard *et al.*, 2011 ▶). Although one-dimensional probe recovery was demonstrated successfully (Kewish *et al.*, 2010 ▶), for imaging two- or three-dimensional objects, the lack of redundant information in the horizontal direction results in an incomplete and inaccurate recovery of the probe, which could cause unexpected electron density variation in reconstructed images.

The manner of obtaining three-dimensional images with Bragg CDI differs from ptychography with transmission geometry (Dierolf *et al.*, 2010 ▶), where the sample was rotated and the two-dimensional ptychographical measurement was repeated at each rotation angle over a full angular range of 180°. In the reconstruction process, two-dimensional images for all rotation angles are reconstructed first, and then the three-dimensional image is obtained through back-projection tomography. With our Bragg reflection geometry, the reverse order was used: a three-dimensional diffraction pattern was collected at one scan position before the sample was translated to the next spot. Typically, a 1° rotation range was sufficient to cover the entire three-dimensional diffraction pattern, simplifying the data acquisition process.

When performing a two-dimensional ptychographical scan on an extended object, the illuminated sample volume is defined by the size and shape of the incident beam. In the rocking-curve three-dimensional diffraction data acquisition process of Bragg CDI, since the sample rotates relative to the beam, the sample boundaries determined by the illumination vary for different angles, which introduces a softly defined sample volume. The collected three-dimensional diffraction pattern could be blurred out, because each data frame is from a slightly different sample volume. Considering a 500 nm-thick sample with 1° rocking-curve measurement, the non-rigidly defined boundary range is about 9 nm. Although this is smaller than the currently achieved spatial resolution, its influence on diffraction data and how it affects reconstructions are unknown. We avoided this potential problem by using the ZnO arm extended only in one direction and performing a one-dimensional ptychographical scan to ensure that each three-dimensional diffraction data set is from the same sample section.

In this study, we were able to treat the probe as a two-dimensional complex wavefront, because the depth of focus of the KB mirrors is much larger than the sample thickness. In other cases with sharply converging focus or where the sample thickness is comparable to the depth of focus, the variation of the beam profile inside the sample is not negligible, and the probe has to be treated as a three-dimensional complex wavefront. It has been proved by numerical simulation that adding a rotational degree of freedom in the regular translational ptychographical scan sequence can recover the probe’s structure in the third dimension (Hruszkewycz *et al.*, 2011 ▶), where it is noticeable that the reconstructed crystal magnitude is very uniform once the three-dimensional probe is recovered. This might suggest that the two-dimensional approximation of the probe is another source for the ‘hot spots’.

## 5. Conclusion

We have constructed a three-dimensional complex image showing the electron density and strain field of a ZnO microstructure with extended size in one direction, using Bragg coherent diffraction imaging combined with the ptychography method. The vertically standing 2.5 µm-long ZnO crystalline sample was scanned through a 1.3 µm focused X-ray beam along the vertical direction in six steps with step size finer than 0.5 µm. A three-dimensional diffraction pattern in the vicinity of the 101 Bragg peak was collected at each scanning position. The overlapped sample section that was illuminated by two successive scans defines a new constraint for the diffraction pattern, which removes the limitation of sample sizes in regular CDI. The dimensions of the obtained image agrees well with SEM measurements.

The redundant information from overlapped scans also allows us to recover the complex wavefront of the incident X-ray beam. We observed a faint phase curvature in the beam and found that the vertical focus was located about 4.04 mm downstream of the sample plane. Since the measurement was a one-dimensional ptychographical scan in the vertical direction, the recovered probe only contains partial horizontal information. A partial coherence analysis was implemented in the image reconstruction process to accommodate imperfect coherence of the beam. This analysis shows that the incident X-ray is fully coherent in the vertical direction and the longitudinal coherence length is about 720 nm, both of which are consistent with expectation or previous measurement.
